# Characterising the performance of a drone-mounted real-time methane imaging system

**DOI:** 10.1038/s41598-025-93186-z

**Published:** 2025-03-13

**Authors:** Angus G. MacGruer, Steven D. Johnson, Simon P. Mekhail, Kyle J. Nutt, Miles J. Padgett, Graham M. Gibson

**Affiliations:** 1 School of Physics and Astronomy, University of Glasgow, Glasgow, G12 8QQ UK; 2 Department of Physics, University of Strathclyde, Glasgow, G4 0NG UK

**Keywords:** Environmental impact, Optical sensors, Diode lasers, Imaging techniques, Natural gas

## Abstract

We demonstrate a methane gas imaging system mounted to an unmanned aerial vehicle (UAV) that is shown to perform real-time detection at distances up to 10m whist airborne. Laser diodes that switch between on- and off- resonance with a ${1.6}{{\upmu \hbox {m}}}$ methane absorption line are used to flood-illuminate a scene. The scene is imaged with an infrared InGaAs camera and the differential of the on-resonance and off-resonance back-scatter images are used to reveal the gas distribution. The performance of the system was characterised against a range of back-scatter surfaces, showing promising applicability to realistic gas sensing environments. We demonstrate that the system is capable of detecting a gas concentration of 5000 ppm.metre up to a range of 13.6m.

## Introduction

Gas leaks are a contributor to global warming due to methane being a powerful greenhouse gas, especially in terms of immediate short term impact^1,2^. Methane is widely used as a key component of natural gas, with leaks potentially having great economic repercussions^3,4^. Previously, the standard detection protocol relied upon detectors that work at a single point, while these can give accurate readings of gas concentration they do not provide an imaging component, making detection of the source of leaks difficult^5^. We present a stand-off method of gas detection where video of the gas location is produced in real-time, thereby developing a tool for much faster and efficient leak identification.

Stand-off detection of gases can be performed via optical absorption, these techniques include tunable diode-laser spectroscopy (TDLAS)^6^ and differential absorption Lidar (DIAL)^7,8^. In general such systems are focused upon accurately measuring the concentration of the gas rather than displaying a live image of the gas distribution, and also require a level of calibration^8–10^. While the system discussed in this paper does not provide precise concentration measurements, it does display a live image of a gas distribution, allowing the source of leaks to be located. Our system, and the other optical absorption detection, techniques can be termed “active” in that they illuminate with a light source of a wavelength tuned to an absorption band of the gas. As an alternative “passive” systems are also possible but these are dependent on environmental factors such as natural background illumination, temperature, wind speed and wind direction in order to identify the gas source^10–15^.

Perhaps the most obvious comparison to our system is that of optical gas imaging (OGI) hardware. These systems are widely used, commercially available and effective at highlighting gas leak locations. The current best-standard OGI devices are passive sensors, operating at wavelengths in the mid- or long-wave infrared, wherein they use specific filtering in order to highlight temperature differences where gas is present^16,17^. This results in a thermal image in which the presence of gas is depicted by a black region or cloud, a result of absorption at the filtered wavelength^18^. This can often result in a need for cooling the sensor which can drive up the prices for such systems^10^. The major drawback to the system is that because the filters used are not specific enough to individual gas absorption lines it can mean that identification between different gas species with similar absorption properties can be difficult^16,17^. This lack of specificity does however, allow more than one gas species to be imaged using only one system, which adds to the versatility of such systems^17^. Similarly, it can be difficult to measure concentration of the gas imaged due to these issues^17^.

Previous developments of an imaging system were able to give some indication of gas distribution from a static location^19^, but there were difficulties in matching the position within the infrared detection camera and a visible camera to enable to operator to pin-point the relative position of the gas distribution in a moving system. Reducing parallax in the system and developments in the image processing to account for motion artefacts have enabled a system more resilient to motion to be produced^20^. The detection system is entirely self contained via the addition of an embedded mini-PC, touch screen and rechargeable lithium ion-based power supply. The system can additionally be mounted to a drone for use within the field^21^.

One particular application envisaged is the inspection of gas pipelines. However, pipelines can lay along or below a wide variety of surfaces, with potentially different absorption and scattering properties. In order for the system to be a viable technology for detecting gas emissions, it must be able to image gas against a wide variety of common background materials and scenes. Traditional techniques for surveying the position of a gas leak can be conducted on emulated surfaces such as grass or paving, something the system must be robust against^22^. Pipelines themselves are metallic in nature, so metal surfaces must be able to return adequate signal.

The key focus of our research is to test the validity of gas leak localisation when conducting a drone-mounted survey. The desire to drone-mount primarily comes from a user safety perspective, methane gas leaks can be hazardous due to their associated explosion risk^23^. In addition to improving user safety, a drone offers the promise of a higher degree of automation and accessibility, potentially leading to an overall increase in regularity of routine facility inspections leading to improved leak detection timescales^23,24^. In addition to being able to follow pre-determined routes, drone swarms can be deployed to quickly cover large areas in fine detail^24,25^. Drones offer an increased degree of flexibility in comparison to helicopter based instruments, due to decreased size, cost of running, improved manoeuvrability in confined or complex spaces, lower flight height, and decreased interference from down drafts caused by the helicopter rotors^26,27^. Drones can also be deployed with ease in less built up areas where access via vehicle is limited^28^.

An issue that presents itself with drone usage is the associated limited flight time leading to relatively short survey duration^26^. Whilst having less impact than helicopters, drones do still generate some downwards flow of air which can disrupt and disperse gas below the detection limit^29^. In order to circumvent this issue instruments are often pointed at an angle ahead of the drone where the downwards flow does not disrupt the gas cloud^30,31^. Depending on mounting type it can be difficult to fully dampen the impact of drone vibrations^23^. Even in a relatively stable hover mode, vibrations caused by the rotors can still result in motion artefacts and hence reduced detection efficiency^23^. The introduction of compensatory technology such as a gimbal has been shown to aid in reducing the impact of these vibrations^32^. Despite these issues, there are reports of gas detection instruments being successfully integrated onto drone systems^30,33^.

We introduce our gas imaging device and present the developments made to the system and the image processing procedure used for methane detection. There have been significant improvements in the gas detection performance and these are discussed. To emulate the end-user application field trials have been performed outside of the laboratory, where the device was mounted to a UAV and tested by looking for methane filled balloons using real-time image processing. The system was also tested against a variety of common surfaces at a range of distance to investigate how universally applicable it would be in real world scenarios.

## Gas imaging hardware

**Fig. 1:**
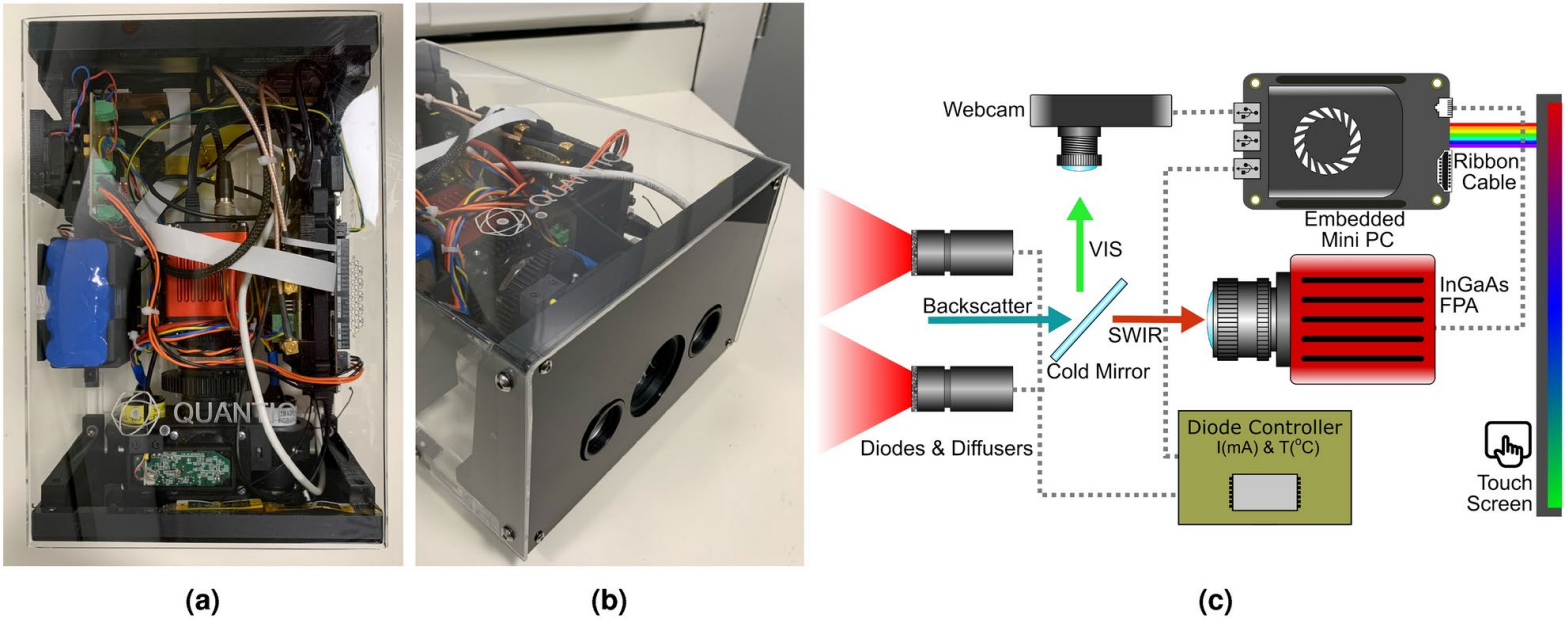
Configuration of the gas imaging instrument. (**a**) Photograph of the completed instrument from above showing the internal configuration of the components. (**b**) The illumination and detection apertures. (**c**) A schematic representation highlighting the main components, namely the webcam, SWIR FPA, SWIR diodes, cold mirror, engineered diffusers, embedded mini-PC, diode control circuitry and touch control screen.

There are four main components to the imaging system, a $256\times 320$ InGaAs short-wave infrared (SWIR) focal plane array (FPA) running with an exposure time of 20ms, a visible camera, and two $\approx {10}{\textrm{mW}}$ InGaAs laser diodes. The diodes are tuned to the absorption wavelength of methane at ${1.653}{{\upmu \hbox {m}}}$ and diffused through ${20}^{\circ }$ engineered diffusers, which provide an expanded, uniform, square illumination profile. The camera also features a band-pass filter centred on ${1.6532}{{\upmu \hbox {m}}}$ and has a full width at half maximum (FWHM) of 18.8nm in order to block unwanted background light. The system itself has a mass of ${3.15}{\textrm{kg}}$, and a size of ${284}{\textrm{mm}}\times {198}{\textrm{mm}}\times {125}{\textrm{mm}}.$ The embedded lithium ion battery source provides more than 2 hours of continuous run time. An image of the system and simplified schematic are presented in Fig. 1. The illumination light will pass through a scene before being back-reflected by a background surface towards the entrance aperture. Upon reaching the entrance aperture a cold mirror will separate incoming light into its visible and SWIR components towards the respective camera detectors.

An on-resonance image will be taken, where methane present in the scene will absorb the illumination light and not be back-scattered. Immediately after this, an off-resonance image will be taken, where the illumination light is slightly detuned via current and temperature control such that it is no longer absorbed by the gas. The fact that the diodes are temperature controlled, both with tuned temperatures of around 22-$25^{\circ }$C, means that the system is overall relatively robust to external temperature changes. The system is designed such that there is air flow throughout to prevent internal temperature from rising too high. The detuning is achieved by decreasing the input voltage to the diodes such that no significant absorption was observed. This introduces a slight power discrepancy between the two frames, however, as the off resonance has marginally less laser power this acts to slightly suppress the background when subtraction is performed. For efficiency the direction of tuning, and hence subtraction, is swapped with each iteration to increase the frame rate. The overall system is controlled by an in-built mini-PC which can be operated using an embedded touch display. The mini-PC also supports a HDMI output which can allow for integration to larger displays as well as to the streaming channel of the drone. This allows the feed to be monitored in real time whilst the system is airborne.

## Image processing

The two captured images are smoothed using a Gaussian filter and convolved with an $8\times 8$ array of ones in order to smooth the response. A correlation is performed in the system to overlap the consecutive frames before subtraction. The correlation used for the data in this paper involves passing the image through the subsequent frame vertically and then horizontally by 20 pixels each time and looking for a local minima in difference between the two frames. Other techniques such as phase correlation have also been experimented with, however this method was both fast and robust for our purposes^20^. The images are then subtracted to produce a differential signal image. The differential image is always reported positive, as the sign of the subtraction is swapped for every odd frame. It follows that any negative signal in the differential image is background noise and so can be removed. The differential signal is, as such, subject to the constraint that if the differential signal is negative, it can be set to zero. The differential image can now be used to generate a binary mask, where a value of 1 denotes a potential presence of gas at the given pixel. The following issue is that, although much of the noise is removed at this point, some background noise is positive and will appear in the binary mask. Steps must now be taken in order to effectively tell the difference between real gas signal and noise. A further threshold constraint cannot be utilised here because at larger distances the noise and true signal are often comparable in magnitude. Instead the signal is distinguished from noise in terms of spatial extent and persistence in time.

False signals caused by background variation rarely appear in the same location in consecutive frames, whereas a real signal mostly will. As such the mask generated in the previous frame is multiplied by that of the current frame performing a pixel-wise AND operation. In each of the data sets analysed in the following a harsh persistency filter was used for consistency, this involved ensuring that data was located in the same position for six consecutive frames to be considered a true signal. This is very effective in stationary systems observing gas cells or balloons, but is less consistent where there is moving or evolving signal such as a gas leak or a moving scene such as that acquired from a drone in motion. As a result there will be a dulling in the sensitivity of the drone data, which may lend itself to an increased uncertainty. Though the vibrational motion of the drone’s rotors in hover mode is too small to have much impact, slight gusts of winds and other movement of the drone itself may temporarily result in this dulling. To filter by spatial extent erosion and dilation are used on the mask after the persistency filter is applied. The size and extent of the erosion and dilation can be varied depending on usage, an erosion kernel of size 3x3 was selected. The kernel itself is binary and comprised of 5 ones and 4 zeroes, with the zeroes being positioned in the corners of the kernel. The kernel moves along the image by centring on each pixel and exploring the nearest neighbours. The kernel acts based upon an OR operation in which, for the erode, if a one in the overlaying kernel overlaps with any zero in the mask then the pixel being investigated is set to zero. For the dilate step if a one in the kernel overlaps any one in the mask then the investigated pixel is set to one. The edges of the image are examined by applying a temporary border of ones to the image to allow the process to occur. This erosion kernel cannot be too large as at increased ranges the signal can be comprised of relatively few pixels, however, as the persistency filter has already been applied a small kernel works sufficiently well to reduce false positives in the mask.

**Fig. 2:**
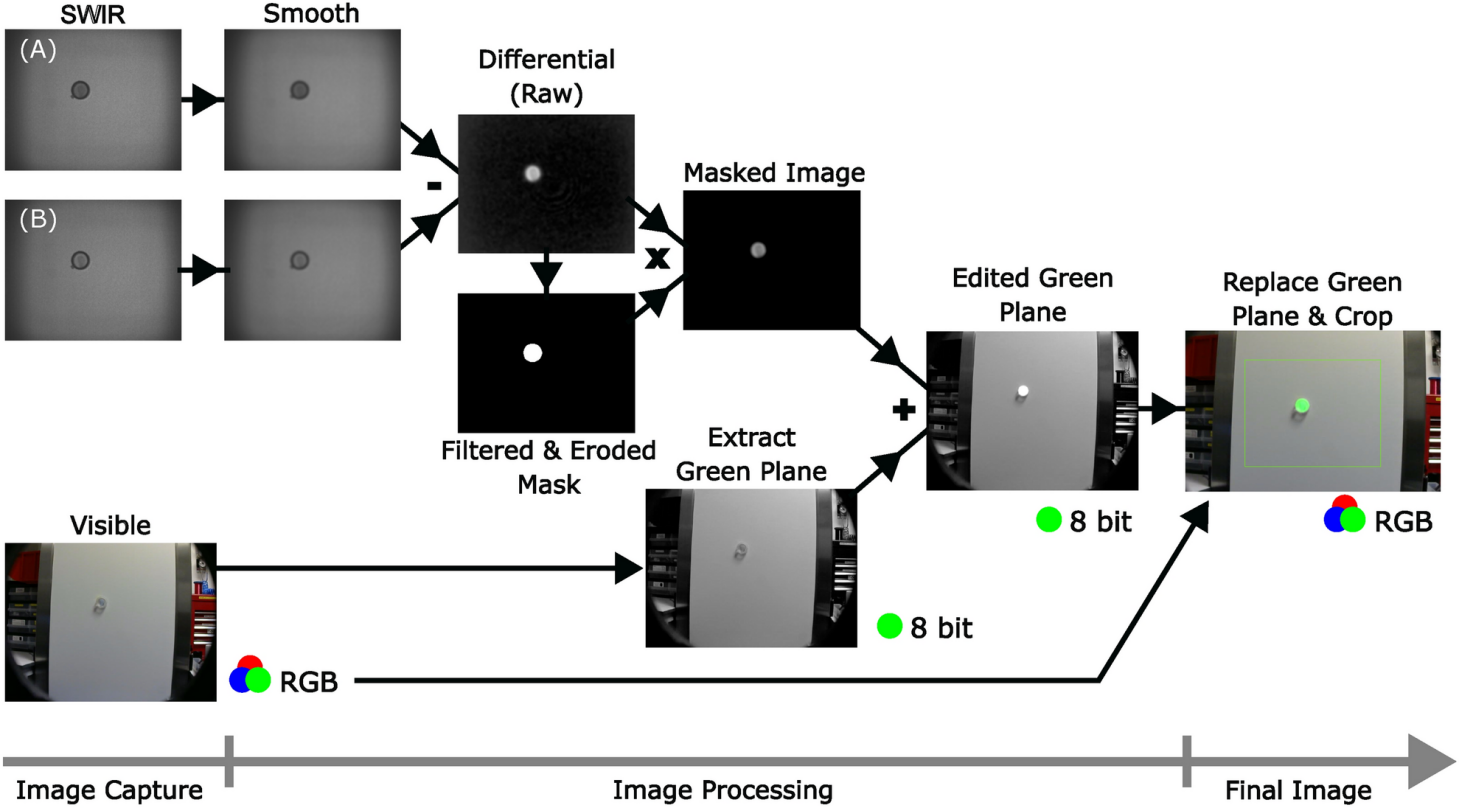
Visual timeline of the image processing pipeline. Smoothing consists firstly of a Gaussian blur followed by a general flattening via convolutions with a $8\times 8$ array of ones. A mask is generated from the differential image and filtered and eroded before being reapplied to the differential image. The masked differential SWIR image is added to the green channel of the visible RGB feed. This conversion can lose some of the magnitude data but will not subtract from the positional data, which is the primary function of our imaging system.

After filtering, the mask is applied to the original differential image to extract the gas positional data. The masked differential image is then formatted from 16-bit to 8-bit and added to the green channel of the captured webcam feed of the scene. This provides a false colouration for methane and reveals the location of the gas signature in the frame. A timeline of the image processing is presented in Fig. 2. The raw SWIR and visible images can be saved for post processing later. Additionally, for the frames presented throughout this paper a further Gaussian filter and linear multiplicative gain is applied to the signal to enhance clarity. In the general use case of the camera this linear gain can be controlled in real time on the touch screen. These aesthetic steps are not included in further signal and noise processing.

In post processing an average signal strength is calculated by multiplying the differential signal by the mask, summing over all pixels, and dividing the result by the sum of the mask over all pixels. An overall average signal for an observation is found by averaging the signal strength over all frames in the observation. In order to quantify uncertainty on this measurement a standard deviation value is calculated for each average signal. This standard deviation will encapsulate variations in signal strength. To allow the persistency filter to take effect the initial few frames are not used when calculating this average. This process is used to characterise the system.

**Fig. 3:**
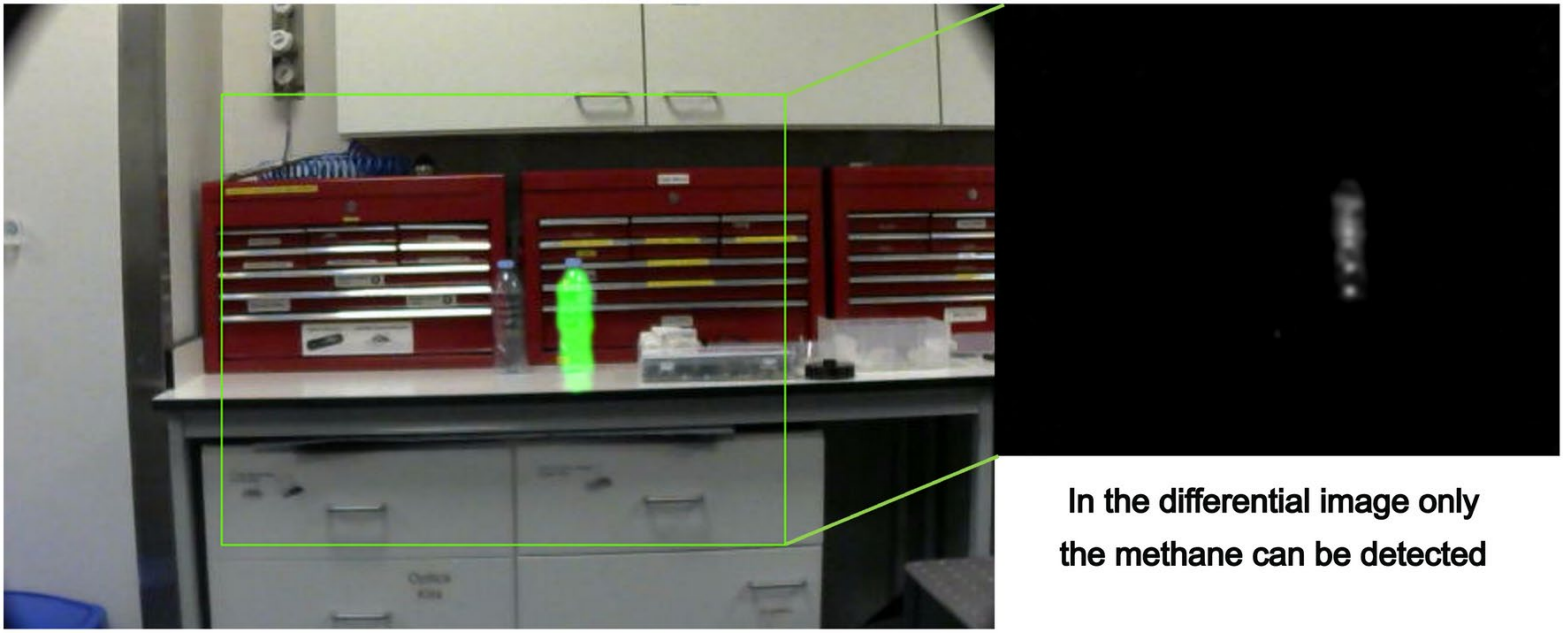
Image taken using the gas imaging device of two clear bottles, one containing butane and the other containing methane. It can clearly be observed that only the methane bottle returns the bright green gas signature, highlighting the specificity of the absorption band selected for investigation. This is further highlighted in the insert which shows the processed differential SWIR image, where signal is only present for in the region of methane gas.

The fact that the diodes are tuned to a specific absorption wavelength of methane means that it has a high level of specificity, escaping a key shortcoming of other, passive, OGI cameras. The effectiveness of this is highlighted in Fig. 3, wherein we detect no signal from other similar gas species.

## Drone campaign

**Fig. 4:**
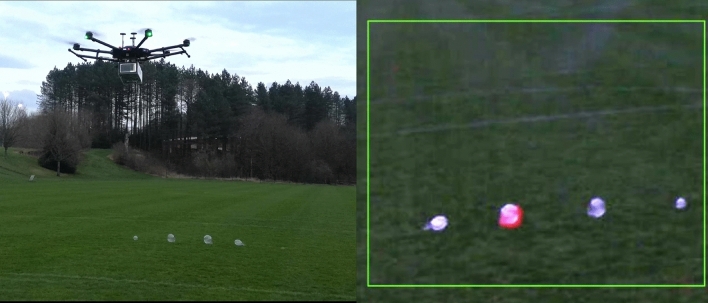
The drone-mounted methane imaging system. Left image depicts the system in flight, while imaging 4 target balloons. Right image is an example frame observed from the gas imaging system from a vertical height of 3m during a fly in shot from a horizontal distance of 22m to 10m at a background light level of 52Lux. The methane filled balloon is highlighted in red in contrast to the three containing nitrogen. The methane is coloured red here to distinguish more clearly from the grass background. Due to low light intensity a $3\times$ gain is applied to the visible feed to increase clarity. This is an example frame from a video attached as supplementary material S1.

The system was attached to the underside of a drone (DJI Matrice 600-Pro), an HDMI connection between the drone and the device meant that a live stream of the camera data could be relayed to the mobile device controlling the flight, an image of the combined system can be observed in Fig. 4. We opted not to implement a more sophisticated motion compensating gimbal system due to the increased mass and the custom nature of our system’s mounting. The maximum possible battery life of the drone without a load is estimated to be around 30 minutes, significantly less than the maximum battery life of the system, making the gas imaging system not the limiting factor on acquisition duration. Four gas balloons were observed from a fixed horizontal range of 10m from the balloons and an altitude of 3m. This altitude was selected as it was deemed a reasonable height from the ground where the effects of rotor down-wash do not interfere strongly with the balloons. This height also ensures that the horizontal range of the system remains reasonable, meaning that the down-wash impact was diminished further. A height of 3m, which provides an approximate horizontal detection range of 10m, should be acceptable for gas pipeline observations. Of the four balloons three contained nitrogen, which cannot be detected by the system, with the other containing methane. A series of images were captured of these balloons from the drone as the evening progressed.

The drone mounted combined system was trialled during times at which sunlight levels were low, namely during sunset. The lux levels were recorded on a light exposure meter (RS Pro ILM-201L). The lux ranged from roughly 400Lux to as low as around 10Lux, corresponding to values for sunset and into the night^34^. The results acquired around sunset were favourable when compared to those acquired during high sunlight levels, as it was previously discovered that in conditions of moderate sunlight the system suffers a large loss of sensitivity. The reason for this is that the SWIR camera will quickly saturate due to sunlight passing our filter, however narrowing our filter greatly impacts the field of view of the detector, as such we gather our data below 400Lux^20^. Additionally, a benefit of working at night is that we can avoid variation in the absorption profile of methane as a result of temperature. Higher temperatures can lead to a decrease in absorption from the gas, which can negatively impact quantitative evaluation of the concentration of gas present, though for significant impact in accuracy the temperature would have to vary considerably^35,36^. Sunlight levels were also not so low that there was a lack of light for visible camera operation, such as during nighttime observations.

## Background testing

**Fig. 5:**
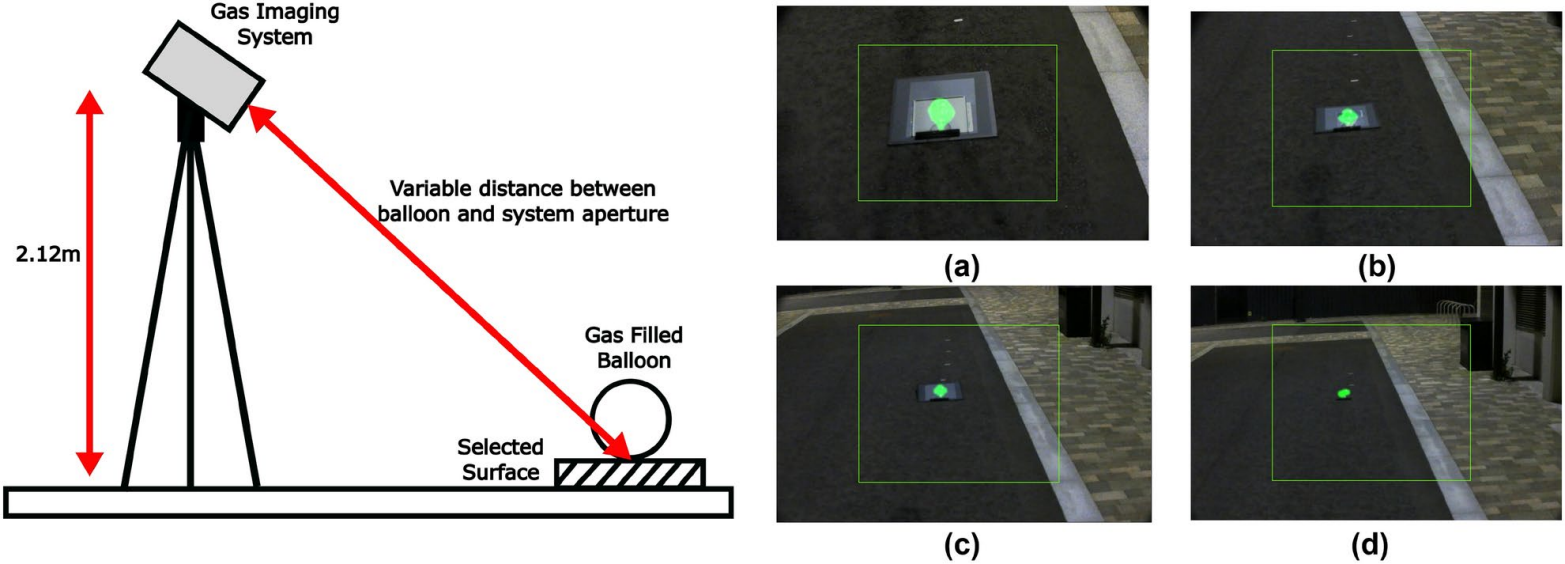
Simplified schematic for surface investigation accompanied by example images taken by the device for (**a**) steel at a distance of 3.1m, (**b**) aluminium at a distance of 5m, (**c**) $18\%$ reflective photography calibration card at a distance of 7.1m, and (**d**) tarmac at a distance of 9m. Note that a gain of 0.7 was applied to the visible channel to enhance clarity of the gas signal in these frames.

**Fig. 6:**
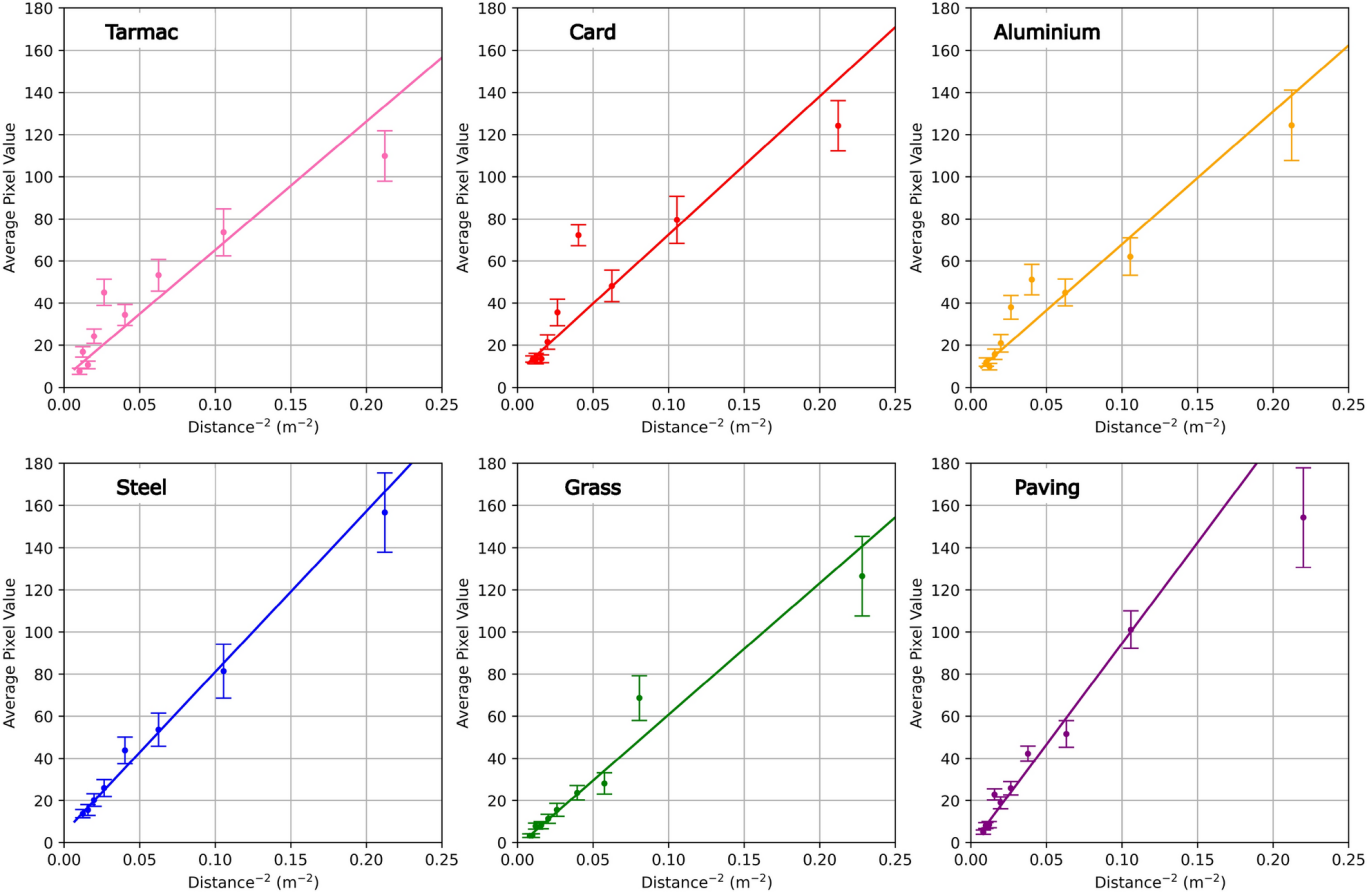
Linear fits of the returned gas signal from the balloon as a function of distance (to the power negative two) on tarmac, card, aluminium, steel, grass, and paving surfaces.

Due to battery life and location constraints for drone flight, testing could only be performed with grass as the background surface. For end-user applications, testing was performed against a wide range of background surfaces each with different scattering profiles. In order to characterise performance against these profiles the gas imaging instrument was set up on tall tripod and taken to a variety of different locations with different surfaces to emulate the data from the drone, examples are shown in Fig. 5. The surfaces explored were grass, paving stone, tarmac, steel and aluminium. Additionally, an $18\%$ grey calibration card, commonly utilised in photography, was used as a reference frame for reproducibility. The tripod was then moved away from the balloon until it reached a distance comparable to that obtained on the drone. The strength of the gas signal as a function of distance was then plotted for each of the explored surfaces to allow for direct comparison. These observations were conducted over the course of two evenings, with grass and paving stone investigated on the first evening and tarmac, card, steel, and aluminium investigated on the second. The distance to the balloon from the aperture of the device was measured each time and the signal levels calculated, these are plotted in Fig. 6. For the card, steel, and aluminium the balloon was placed atop a sheet of the respective material and it was checked by eye that the light the camera detects has passed through the balloon and reflected off of the test material as opposed to underlying tarmac. In some cases for increased distances, this involved introducing a tilt in the test material by propping it up. In order to avoid the impact of sunlight issues the data was collected after the sun had set. As this is a stationary system, the correlation aspect of the processing was not implemented to optimise system speed.

The main issue the system faced was unwanted sunlight signal, which under some weather conditions could saturate the camera. This interference arises from the fact that our current filter does not block enough of the incident sunlight and as such more was detected by the camera than intended. The details of this are explored in more detail in previous publications^20,21^, but is summarised briefly here. In order to reduce incident sunlight on our SWIR camera the filter in front of the camera must be a narrow band-pass, however reducing the FWHM of the filter also decreases the angular field of view as a consequence of the tilt-tuning of band-pass interference filters^20,37^. As back scattered light is incident from a variety of angles, narrow filters in this scenario result in a reduction in field of view, limiting the full frame capabilities of the system. The combined drone mounted system was also susceptible to a higher level of noise in addition to that caused by sunlight. The movement of the drone caused small amounts of noise, mainly in regions of the image with non-uniform texture such as grass, much of this could be addressed with the computational filtering as outlined in this work. However, this could lead to an overall decrease in sensitivity. Overall, the effects of sunlight upon the gas detection protocol is complex and whilst some attempts have been made to reduce its impact on the data, this is a continuing area of research.

As shown in Fig. 6 we can see that the signal follows an inverse square relation with distance, which is to be expected as a result of the imaging mechanism. The light leaving the diodes must be diffused, travel through the gas, scatter off the background and be collected by the camera. This causes much of the light intensity to be lost. This loss follows an inverse square relation with distance and as such it is expected the return signal from the gas would also follow this trend. What is also evident from this data is that in general no surface performs significantly worse than another. Whilst initial return signals are varied, with the strongest returns from paving and steel and the weakest from tarmac, all six surfaces begin to perform similarly at further distances and, most importantly, all return clearly identifiable signal as the distance begins to approach 10m. As such it can be observed that the system can be used in a variety of real world scenarios with no great detriment to overall response. We observed an increased uncertainty on the surface return signals closer to the camera than those further away. It is believed that the reason for this was a result of fluctuation in size of the signal mask. The mask is inherently larger for larger signals and as such fluctuations around the circumference of the mask lead to larger variation in the signal value than with a smaller circumference.

**Fig. 7:**
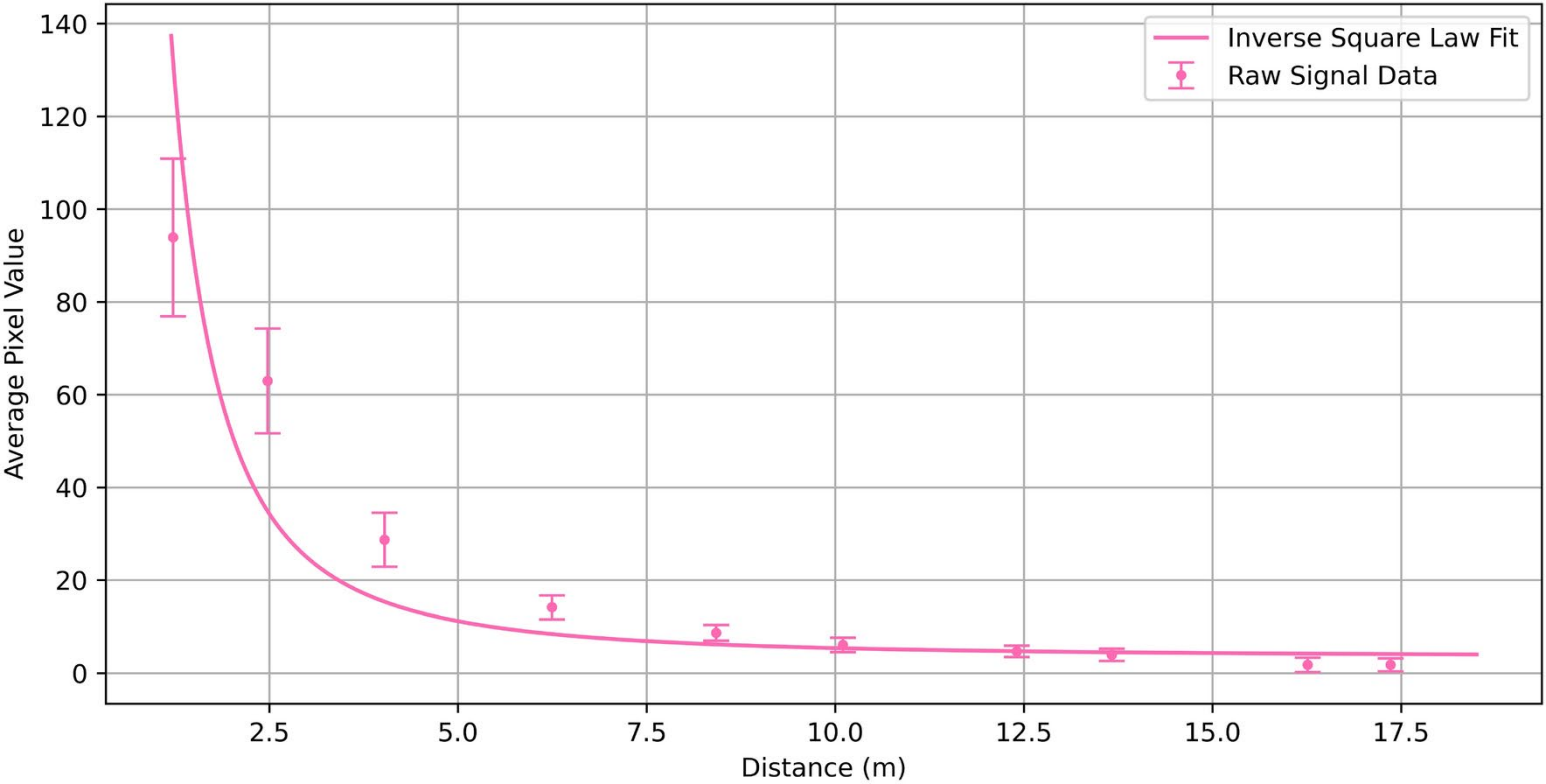
Return signal from a methane cell containing 5000 ppm.metre of gas. It can be seen to follow a similar trend to the surface data. The signal and noise become similar in size for the data points at 16.3m and 17.3m, what this results in is a gas signature that will occasionally flicker in and out. As such we can say that the threshold for reliable detection of a gas signature is approximately 13.6m where this is not the case.

As the gas cells used in these trials were unquantified and used primarily for demonstration of the stability of the system, we also have performed a trial on a gas cell containing a measured amount of gas to place the system response in wider context. The cell used contains an approximate maximum concentration of 5000 parts per million metres (ppm.metre). The cell was placed in front of a wall and the camera moved away from the cell as in the surface tests. The results of this experiment are presented in Fig. 7. The data was processed using the same methodology as for the surfaces. This data suggests that the system is capable of detecting 5000 ppm.metre of gas up to a range of around 13.6m. It can be seen that, as in the surface data, the return signal increases in an inverse-square relation with distance as a result we imagine that the sensitivity to concentration would behave in a similar trend, meaning that lower concentration could be observed at a closer range and higher concentrations at further distances. In order to fully characterise this response one would need to survey a wide range of cells at different concentrations, which is outside the scope of this paper.

## Discussion and conclusions

We have presented a real-time methane imaging system with wide applicability in the field. By highlighting the system’s ability to be drone mounted we demonstrate its flexibility as a device that can be used handheld by a user or controlled remotely to access different dangerous or difficult locations. We additionally indicate the systems resilience to different background surfaces, concluding that at ranges of up to 10m return signal is always obtained such that gas location can be identified. This level of robustness in regards to the back-scatter surface profile also further highlights the potential widespread use of the device in pipeline surveys, where such pipelines could be located under streets or alongside grass. We provide additional context for the systems performance by characterising the signal response as a function of distance for a cell containing 5000 ppm.metre of gas, demonstrating sensitivity up to approximately 13.6m. Further work with the system will seek to improve performance in direct sunlight, further increasing the wide applicability of the system as a monitoring device.

## Methods

The two images were captured using a SWIR camera (Goldeye G-008 InGaAs SWIR FPA) running with an exposure time of 20ms with a Kowa LM25HC-SW 25mm C-mount lens attached, and a visible camera (Logitech c270 webcam) running at 30 frames per second with a 4mm lens. The two $\approx {10}{\textrm{mW}}$ InGaAs laser diodes (Eblana Photonics EP1653-7-DM-TP39-01) were tuned to the absorption wavelength of methane at ${1.653}{{\upmu \hbox {m}}}$. Each diode was controlled using a custom circuit board mounted with a Thorlabs MLD203CLN current controller chip and a MTD415T temperature controller chip. The base current and temperature was set and calibrated using Thorlabs propriety software and finely tuned using the analogue input pin of the current control chip via an Arduino Nano and an Analogue Systems AD5696 DAC. The laser diode outputs were collimated using 30mm lenses and passed through Thorlabs ED1-S20-MD ${20}^{\circ }$ engineered square diffusers. The back-scattered diode light passed through a cold mirror (50.8mm Thorlabs FM203 mirror) before reaching the respective cameras. A band-pass filter (Spectrogon) centred on ${1.6532}{{\upmu \hbox {m}}}$ featuring a full width at half maximum (FWHM) of 18.8nm was used to reduce background signal. A lithium ion battery (Enix Energies MGL2806) charged using a custom circuit board provided a portable power supply. The system was controlled using a mini-PC (Lattepanda Alpha), with accompanying Lattepanda 7” touch display. All components were mounted within an enclosure using custom 3D printed parts and laser cut acrylic base and cover.

## Supplementary Information

Supplementary Information 1.

Supplementary Information 2.

## Data Availability

The datasets generated and analysed during the current study are available in a University of Glasgow Library data repository, http://dx.doi.org/10.5525/gla.researchdata.1697.

## References

[1] Howarth, R. W. A bridge to nowhere: Methane emissions and the greenhouse gas footprint of natural gas. *Energy Science and Engineering* **2**, 47–60. 10.1002/ese3.35 (2014).

[2] Dean, J. F. Old methane and modern climate change. *Science* **367**, 846–848. 10.1126/science.aba8518 (2020). 32079756 10.1126/science.aba8518

[3] Halley, S. & kun Tsui, L. & Garzon, F.,. Combined mixed potential electrochemical sensors and artificial neural networks for the quantification and identification of methane in natural gas emissions monitoring. *Journal of The Electrochemical Society* **168**, 097506. 10.1149/1945-7111/ac2465 (2021).

[4] Alvarez, R. A. et al. Assessment of methane emissions from the U.S. oil and gas supply chain. *Science* **361**, 186–188. 10.1126/science.aar7204 (2018). 29930092 10.1126/science.aar7204 PMC6223263

[5] Well, B. V. et al. An open-path, hand-held laser system for the detection of methane gas. *Journal of Optics A: Pure and Applied Optics* **7**, 10.1088/1464-4258/7/6/025 (2005).

[6] He, Q. et al. A near-infrared acetylene detection system based on a 1.534 m tunable diode laser and a miniature gas chamber. *Infrared Physics and Technology* **75**, 93–99. 10.1016/j.infrared.2016.01.006 (2016).

[7] Browell, E. Differential absorption lidar sensing of ozone. *Proceedings of the IEEE* **77**, 419–432. 10.1109/5.24128 (1989).

[8] Innocenti, F., Robinson, R., Gardiner, T., Finlayson, A. & Connor, A. Differential absorption lidar (DIAL) measurements of landfill methane emissions. *Remote Sensing* **9**, 953. 10.3390/rs9090953 (2017).

[9] Rui-Feng, K. et al. Influence of laser intensity in second-harmonic detection with tunable diode laser multi-pass absorption spectroscopy. *Chinese Physics* **14**, 1904. 10.1088/1009-1963/14/9/040 (2005).

[10] Titchener, J. et al. Single photon lidar gas imagers for practical and widespread continuous methane monitoring. *Applied Energy* **306**, 118086. 10.1016/j.apenergy.2021.118086 (2022).

[11] Maistry, N., Schutz, R. & Cox, E. The investigation of different image processing techniques to improve the visibility of volatile gas plumes in industry using optical gas imaging. 33, 10.1117/12.2501312 (SPIE-Intl Soc Optical Eng, 2019).

[12] Ravikumar, A. P., Wang, J. & Brandt, A. R. Are optical gas imaging technologies effective for methane leak detection?. *Environmental Science and Technology* **51**, 718–724. 10.1021/acs.est.6b03906 (2017). 27936621 10.1021/acs.est.6b03906

[13] Hirst, B. et al. Oil and gas prospecting by ultra-sensitive optical gas detection with inverse gas dispersion modelling. *Geophysical Research Letters* **31**, L12115. 10.1029/2004GL019678 (2004).

[14] Olbrycht, R. & Kaluza, M. Optical gas imaging with uncooled thermal imaging camera - impact of warm filters and elevated background temperature. *IEEE Transactions on Industrial Electronics* **67**, 9824–9832. 10.1109/TIE.2019.2956412 (2020).

[15] Zimmerle, D. *et al.* Detection limits of optical gas imaging for natural gas leak detection in realistic controlled conditions. *Environmental Science & Technology* **54**, 11506–11514, (2020) 10.1021/acs.est.0c01285 . PMID: 32786569, 10.1021/acs.est.0c01285 PMC8193645 32786569

[16] Strahl, T. et al. Methane leak detection by tunable laser spectroscopy and mid-infrared imaging. *Applied optics* **60**, C68–C75. 10.1364/AO.419942 (2021). 34143108 10.1364/AO.419942

[17] Hagen, N. Survey of autonomous gas leak detection and quantification with snapshot infrared spectral imaging. *Journal of Optics* **22**, 103001. 10.1088/2040-8986/abb1cf (2020).

[18] Wang, J. et al. Machine vision for natural gas methane emissions detection using an infrared camera. *Applied Energy* **257**, 113998. 10.1016/j.apenergy.2019.113998 (2020).

[19] Nutt, K. J. et al. Developing a portable gas imaging camera using highly tunable active-illumination and computer vision. *Optics Express* **28**, 18566. 10.1364/oe.389634 (2020). 32672155 10.1364/OE.389634

[20] MacGruer, A.G., Gibson, G.M., Nutt, K.J. & Padgett, M.J. Real-time imaging of methane gas from a UAV mounted system. In *Remote Sensing Technologies and Applications in Urban Environments VIII*, vol. 12735, 127350J, 10.1117/12.2678412. International Society for Optics and Photonics (SPIE, 2023).

[21] MacGruer, A.G., Johnson, S.D., Nutt, K.J., Padgett, M.J. & Gibson, G.M. Drone-based gas leak detection system for use in industry. In *Quantum Technology: Driving Commercialisation of an Enabling Science IV*, vol. 12795, 1279503, 10.1117/12.2691068. International Society for Optics and Photonics (SPIE, 2023).

[22] Cho, Y. *et al.* A closer look at underground natural gas pipeline leaks across the United States. *Elementa: Science of the Anthropocene* **10**, 00095, 10.1525/elementa.2021.00095 (2022). https://online.ucpress.edu/elementa/article-pdf/10/1/00095/747156/elementa.2021.00095.pdf.

[23] Nooralishahi, P., López, F. & Maldague, X. A drone-enabled approach for gas leak detection using optical flow analysis. *Applied Sciences* **11**, 10.3390/app11041412 (2021).

[24] Villa, T., Gonzalez, F., Miljevic, B., Ristovski, Z.D. & Morawska, L. An overview of small unmanned aerial vehicles for air quality measurements: Present applications and future prospectives. *Sensors (Switzerland)* **16**, (2016) 10.3390/s16071072 . 10.3390/s16071072 PMC4969839 27420065

[25] Rossi, M. *et al.* Gas-drone: Portable gas sensing system on UAVs for gas leakage localization. In *SENSORS, 2014 IEEE*, 1431–1434, 10.1109/ICSENS.2014.6985282 (2014).

[26] Emran, B. J., Tannant, D. D. & Najjaran, H. Low-altitude aerial methane concentration mapping. *Remote Sensing* **9**, 823. 10.3390/rs9080823 (2017).

[27] Golston, L.M. *et al.* Lightweight mid-infrared methane sensor for unmanned aerial systems. *Applied Physics B: Lasers and Optics* **123**, 10.1007/s00340-017-6735-6 (2017).

[28] Alvear, O., Zema, N. R., Natalizio, E. & Calafate, C. T. Using UAV-based systems to monitor air pollution in areas with poor accessibility. *Journal of Advanced Transportation* **1–14**, 2017. 10.1155/2017/8204353 (2017).

[29] Sato, R. et al. Detection of gas drifting near the ground by drone hovering over: Using airflow generated by two connected quadcopters. *Sensors* **20**, 1397. 10.3390/s20051397 (2020). 32143359 10.3390/s20051397 PMC7085716

[30] Burgués, J. & Marco, S. Environmental chemical sensing using small drones: A review. *Science of the Total Environment* **748**, 10.1016/j.scitotenv.2020.141172 (2020). 10.1016/j.scitotenv.2020.141172 32805561

[31] Wivou, J. *et al.* Air quality monitoring for sustainable systems via drone based technology. In *2016 IEEE International Conference on Information and Automation for Sustainability (ICIAfS)*, 1–5, 10.1109/ICIAFS.2016.7946542 (2016).

[32] Neumann, P.P. *et al.* Aerial-based gas tomography – from single beams to complex gas distributions. *European Journal of Remote Sensing* **52**, 2–16, 10.1080/22797254.2019.1640078 (2019).

[33] Khan, A. et al. Low power greenhouse gas sensors for unmanned aerial vehicles. *Remote Sensing* **4**, 1355–1368. 10.3390/rs4051355 (2012).

[34] Li, Q. *et al.* Ultra-strong phosphorescence with 48% quantum yield from grinding treated thermal annealed carbon dots and boric acid composite. *SmartMat* **3**, 260–268, 10.1002/smm2.1075 (2022).

[35] Duan, B. et al. Correlation of absorption spectrum properties of methane with coupling variables between temperature and concentration. *Optik* **185**, 82–87. 10.1016/j.ijleo.2019.03.070 (2019).

[36] Nagali, V., Chou, S. I., Baer, D. S., Hanson, R. K. & Segall, J. Tunable diode-laser absorption measurements of methane at elevated temperatures. *Appl. Opt.* **35**, 4026–4032. 10.1364/AO.35.004026 (1996). 21102806 10.1364/AO.35.004026

[37] Löfdahl, M. G., Henriques, V. M. J. & Kiselman, D. A tilted interference filter in a converging beam. *Astronomy and Astrophysics* **533**, A82. 10.1051/0004-6361/201117305 (2011).

