# Supplementary material for: Characterising the performance of a drone-mounted real-time methane imaging system
Source: Sci Rep. 2025 Mar 13;15:8787. doi: 10.1038/s41598-025-93186-z (PMC11906584; doi:10.1038/s41598-025-93186-z)
Supplement: Supplementary file 1 — Supplementary Information 1. [file 41598_2025_93186_MOESM1_ESM.docx]

Supplementary 1 – A video of the drone-mounted methane imaging system. The left-hand feed depicts the system in flight, and is synced to the right-hand feed which shows the direct stream of images from the gas imaging system. The drone reaches a vertical height of 3m and begins at a horizontal distance of 22 m from 4 target balloons, one of which contains methane, before flying inwards to a horizontal distance of 10m. The background light level is 52 lux. Once within range the methane filled balloon is highlighted in red in contrast to the three containing nitrogen. Due to low light intensity a 3× gain is applied to the visible feed to increase clarity. An example frame from this video appears in Figure 4.
